# Time-of-day dependent changes in guinea pig bladder afferent mechano-sensitivity

**DOI:** 10.1038/s41598-021-98831-x

**Published:** 2021-09-29

**Authors:** Stewart Christie, Vladimir Zagorodnyuk

**Affiliations:** Discipline of Human Physiology, Flinders Health & Medical Research Institute, College of Medicine and Public Health, Flinders University, GPO Box 2100, Adelaide, SA Australia

**Keywords:** Neuroscience, Physiology

## Abstract

The voiding of urine has a clear circadian rhythm with increased voiding during active phases and decreased voiding during inactive phases. Bladder spinal afferents play a key role in the regulation of bladder storage and voiding, but it is unknown whether they exhibit themselves a potential circadian rhythm. Therefore, this study aimed to determine the mechano- and chemo- sensitivity of three major bladder afferent classes at two opposite day-night time points. Adult female guinea pigs underwent conscious voiding monitoring and bladder ex vivo single unit extracellular afferent recordings at 0300 h and 1500 h to determine day-night modulation of bladder afferent activity. All guinea pigs voided a higher amount of urine at 1500 h compared to 0300 h. This was due to an increased number of voids at 1500 h. The mechano-sensitivity of low- and high-threshold stretch-sensitive muscular-mucosal bladder afferents to mucosal stroking and stretch was significantly higher at 1500 h compared to 0300 h. Low-threshold stretch-insensitive mucosal afferent sensitivity to stroking was significantly higher at 1500 h compared to 0300 h. Further, the chemosensitivity of mucosal afferents to N-Oleoyl Dopamine (endogenous TRPV1 agonist) was also significantly increased at 1500 h compared to 0300 h. This data indicates that bladder afferents exhibit a significant time-of-day dependent variation in mechano-sensitivity which may influence urine voiding patterns. Further studies across a 24 h period are warranted to reveal potential circadian rhythm modulation of bladder afferent activity.

## Introduction

Circadian rhythms are orchestrated by a transcriptional-translational feedback loop for a set of oscillating ‘clock’ genes (e.g., *Clock*, *Nr1d1*, *Bmal1*, *Per1 *and *2*, and *Cry1* and *2*) with the master clock located in the suprachiasmatic nucleus (SCN) of the hypothalamus^1^. This master clock is influenced by the light–dark cycle and can entrain circadian rhythms in other tissues via hormonal and neural pathways allowing for the coordination of vital bodily processes^2^. It should be noted that peripheral tissues can also contain their own clock, some of which act independently of the SCN^3^.

Voiding patterns in humans^4^ and some mammals such as mice^5,6^ exhibit a circadian rhythm with increased amounts of voiding during the active phases and decreased voiding during inactive phases. Bladder afferents play a key role in controlling both storage and micturition, and are involved in sensations such as filling, fullness, urgency and pain^7–9^. Bladder afferent C and Aδ fibres belong to at least 5 major types of bladder afferent: mucosal, muscular-mucosal, muscular, vascular (serosal), and silent^9–11^. Within these major types, further subdivision into at least 8 classes has been suggested each of which is characterised by the specific structures and locations within the bladder wall and possess specific combinations of ion channels and receptors which regulate their excitability^7,11^. Interestingly, these various classes of bladder afferents respond differently to endogenous stimuli such as endocannabinoids and vanilloids^12^, the receptors of which demonstrate clear circadian rhythms^13,14^.

Other cells of the bladder wall including urothelial and interstitial cells are also involved in modulation of bladder storage and micturition^15,16^ as well as excitability and contractility of the detrusor smooth muscle cells^17^. For example, gap junction proteins (connexins) mediate the propagation of electrical signals through the bladder wall which may significantly influence bladder function^18^. Ion channels that exhibit circadian rhythms such as TRPV4 and Piezo1 on urothelial cells may also influence bladder function via Ca^2+^ influx and subsequent release of ATP^19–21^. All of these may eventually directly or indirectly, via changes in local detrusor contractile activity, modify bladder afferent signalling resulting in changes to bladder capacity and voiding. Therefore, circadian rhythms in micturition may be partially determined by clock genes within the bladder itself controlling bladder function via connexin43^5^, TRPV4^19,20^, TRPV1^22^ and Piezo1^20,21^, all of which demonstrate circadian rhythms. It has been suggested that disruptions in these bladder circadian rhythms may contribute to nocturia^21,23^.

A growing body of evidence indicates the role of circadian rhythms in modulation of visceral afferent activity as seen in the case of gastric vagal afferent mechano-sensitivity, which potentially regulate food intake rhythms^24,25^. However, to date there is no data on circadian rhythm modulation of the mechano- and chemo-sensitivity of the bladder sensory neurons. Therefore, by using in vivo metabolic cage void monitoring and ex vivo single unit extracellular recordings in guinea pigs, the current study aimed to determine whether bladder afferents exhibit time-of-day dependent changes in mechano-sensitivity by studying two opposite day-night time points.

## Results

### Total voiding is higher during the day compared to the night

**Figure 1:**
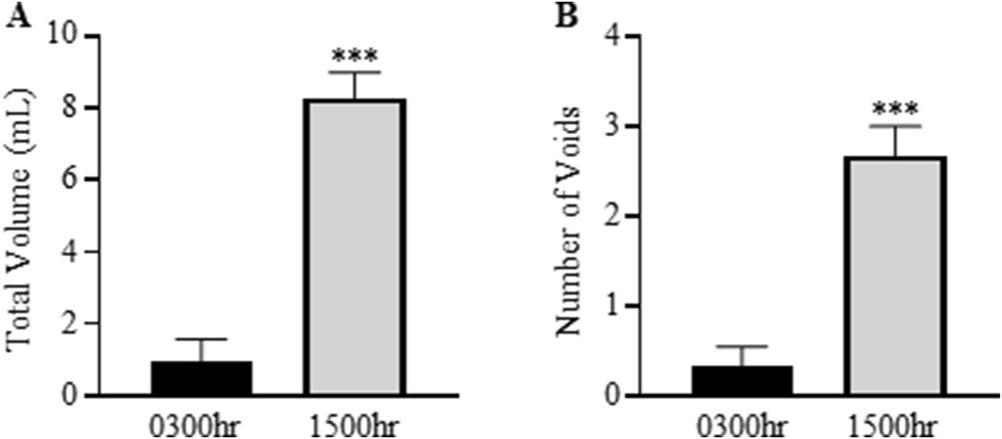
Guinea pigs voided more during the day. (**A**) The total volume voided during 3 h in the metabolic cages at 0300 h and 1500 h. (**B**) Number of voids during 3 h in the metabolic cages at 0300 h and 1500 h. N = 6 guinea pigs for both time points. ***P < 0.001 vs 0300 h.

The effects of time-of-day on voiding patterns is illustrated in Fig. 1. The guinea pigs voided a significantly higher total volume of urine at 1500 h compared to 0300 h (Fig. 1A; 0300 h 0.95 ± 0.61 mL; 1500 h 8.27 ± 0.72 mL; N = 6, P < 0.001). This was reflected by a significantly higher number of voids at 1500 h compared to 0300 h (Fig. 1B; 0300 h 0.3 ± 0.2 voids; 1500 h 2.6 ± 0.3 voids; N = 6 P < 0.001). It should be noted that at 0300 h only 2 of the 6 guinea pigs voided. Of the guinea pigs that did void at both time points, there was no difference between the volume voided (0300 h 2.87 ± 0.37 mL; 1500 h 2.97 ± 0.49 mL).

### Bladder low-threshold muscular mucosal mechano-sensitivity is higher during the day compared to the night

**Figure 2:**
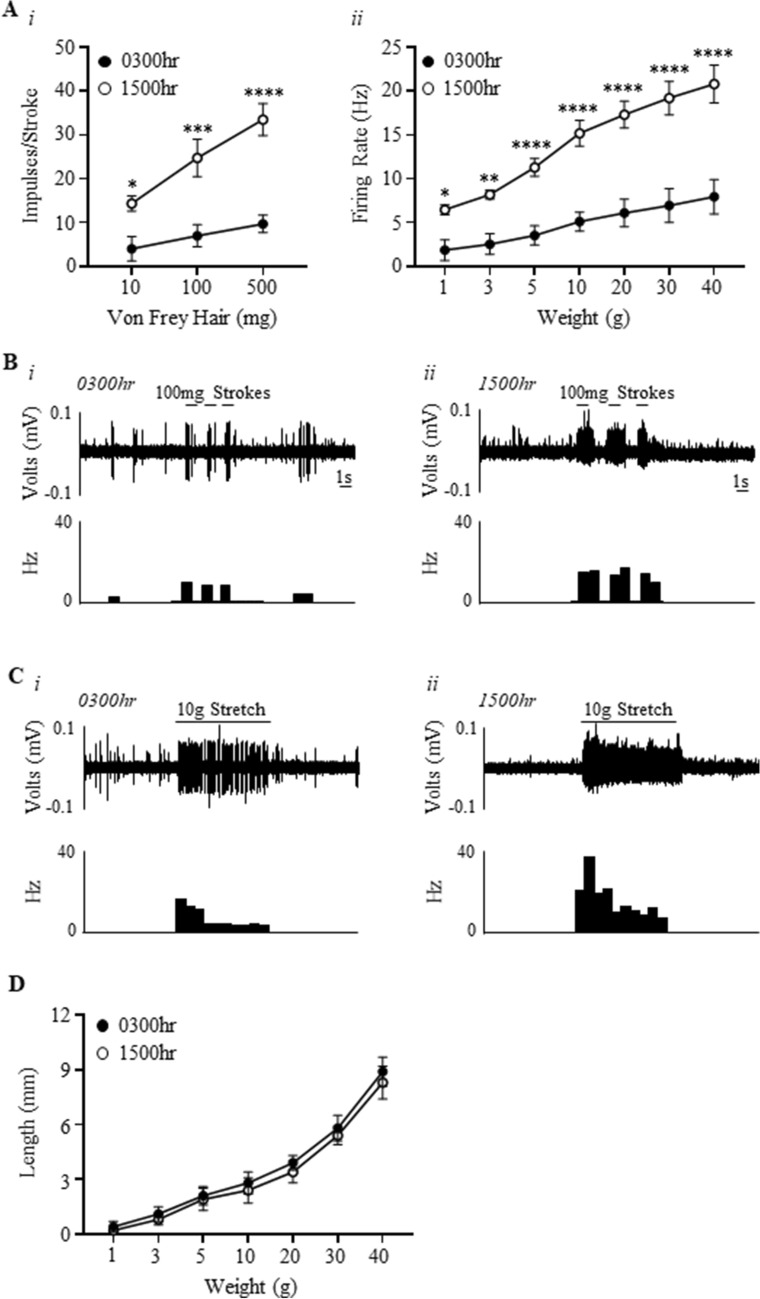
Bladder low-threshold muscular-mucosal afferents are more mechano-sensitive during the day compared to the night. (**A**) The response of low-threshold muscular-mucosal afferents to (*i*) mucosal stroking (10, 100, and 500 mg) and (*ii*) stretch (1–40 g) at 0300 h and 1500 h. N = 6, n = 12 at each time point. *P < 0.05, **P < 0.01, ***P < 0.001, ****P < 0.0001. (**B**) An example of a raw trace of muscular-mucosal afferent responses to 100 mg stroking at 0300 h and 1500 h. (**C**) An example of a raw trace of muscular-mucosal afferent responses to 10 g stretch at 0300 h and 1500 h. (**D**) Bladder compliance determined by measuring length of the bladder preparations during imposed stretch (1–40 g) at 0300 h and 1500 h.

The effects of time of day on low-threshold muscular-mucosal afferent responses to stroking and stretch is illustrated in Fig. 2. Low-threshold muscular-mucosal afferent sensitivity to stroking was significantly higher at 1500 h compared to 0300 h (Fig. 2Ai,B; time-of-day effect (F(1,24) = 71.02, N = 6, n = 12, P < 0.0001), stroking weight effect (F(2,24) = 12.23, P < 0.001), and an interaction (F(2,24) = 3.6, P < 0.05)). The increased mechano-sensitivity at 1500 h was more significant with higher stroke weights (10 mg: 0300 h 4.0 ± 1.2 impulses, 1500 h 14.3 ± 1.7 impulses P < 0.05; 100 mg: 0300 h 7.0 ± 1.1 impulses, 1500 h 24.7 ± 4.2 impulses P < 0.0001; 500 mg: 0300 h 9.7 ± 0.8 impulses, 1500 h 33.5 ± 3.6 impulses P < 0.0001).

Similarly, the responses of low-threshold muscular-mucosal afferents to stretch were significantly higher at 1500 h compared to 0300 h (Fig. 2Aii,C; time-of-day effect, (F(1,56) = 238.1, N = 6, n = 12, P < 0.0001), stretch intensity effect (F(6,56) = 24.52, P < 0.0001), and an interaction (F(6,56) = 4.2, P < 0.001)). Stretch from 5-40 g showed higher levels of significance compared to 1-3 g stretch (1 g, P < 0.05; 3 g, P < 0.01; 5–40 g, P < 0.0001). Bladder wall compliance, measured as the change in length during imposed load (1–40 g) to bladder preparations, was not different between 0300 and 1500 h at all load intensities (Fig. 2D).

### Bladder high-threshold muscular mucosal mechano-sensitivity is higher during the day compared to the night

**Figure 3:**
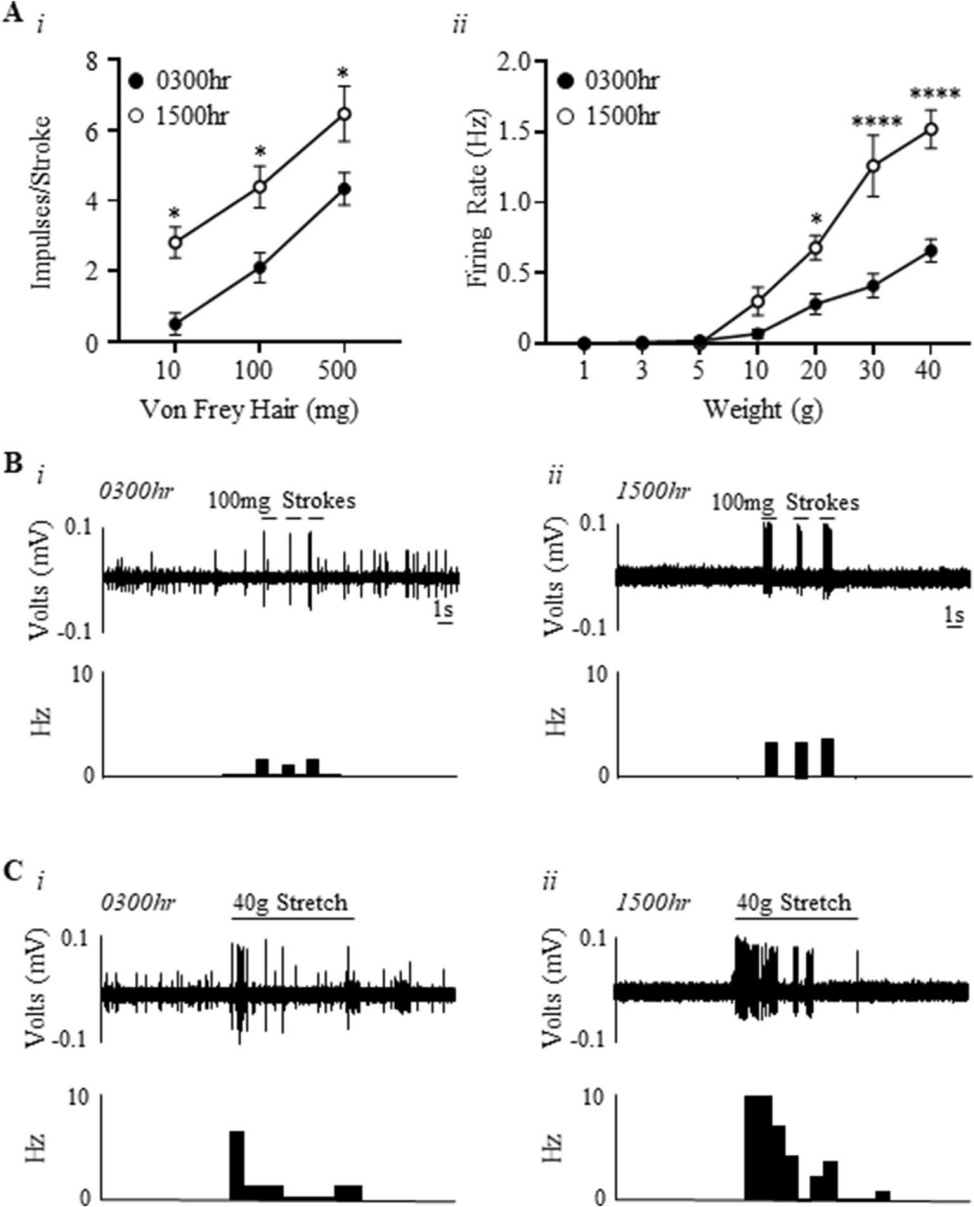
Bladder high-threshold muscular-mucosal afferents are more mechano-sensitive during the day compared to the night. (**A**) The response of high-threshold muscular-mucosal afferents to (*i*) mucosal stroking (10, 100, and 500 mg) and (*ii*) stretch (1–40 g) at 0300 h and 1500 h. N = 6, n = 12 at each time point. *P < 0.05, ****P < 0.0001. (**B**) An example of a raw trace of high-threshold muscular-mucosal afferent responses to 100 mg stroking at 0300 h and 1500 h. (**C**) An example of a raw trace of high-threshold muscular-mucosal afferent responses to 40 g stretch at 0300 h and 1500 h.

Time-of-day dependent effects on high-threshold muscular-mucosal afferent sensitivity to stroking and stretch is illustrated in Fig. 3. The sensitivity of high-threshold muscular mucosal afferents to mucosal stroking was significantly increased at 1500 h compared to 0300 h (Fig. 3Ai,B; time-of-day effect, (F(1,24) = 27.63, N = 6, n = 12, P < 0.0001), stroking weight effect (F(2,24) = 25.7, P < 0.0001), and no interaction). This significance did not increase with increasing stroke weight (10 mg: 0300 h 0.5 ± 0.3 impulses, 1500 h 2.8 ± 0.4 impulses P < 0.01; 100 mg: 0300 h 2.1 ± 0.4 impulses, 1500 h 4.3 ± 0.5 impulses P < 0.01; 500 mg: 0300 h 4.3 ± 0.4 impulses, 1500 h 6.4 ± 0.7 impulses P < 0.05).

The responses of high-threshold muscular-mucosal afferents to 20-40 g stretch were significantly increased at 1500 h compared to 0300 h (Fig. 3Aii,C; time-of-day effect, (F(1,56) = 51.36, N = 6, n = 12, P < 0.0001), stretch intensity effect (F(6,56) = 53.0, P < 0.0001), and an interaction (F(6.56) = 10.25, P < 0.0001). This significance was greater with increasing weights (20 g: 0300 h 0.3 ± 0.07 Hz, 1500 h 0.7 ± 0.08 Hz, P < 0.01; 30 g 0300 h 0.4 ± 0.08 Hz, 1500 h 1.3 ± 0.2 Hz, P < 0.0001; 40 g: 0300 h 0.7 ± 0.08 Hz, 1500 h 1.5 ± 0.1 Hz, P < 0.0001).

### Bladder mucosal afferent mechano-sensitivity is higher during the day compared to the night

**Figure 4:**
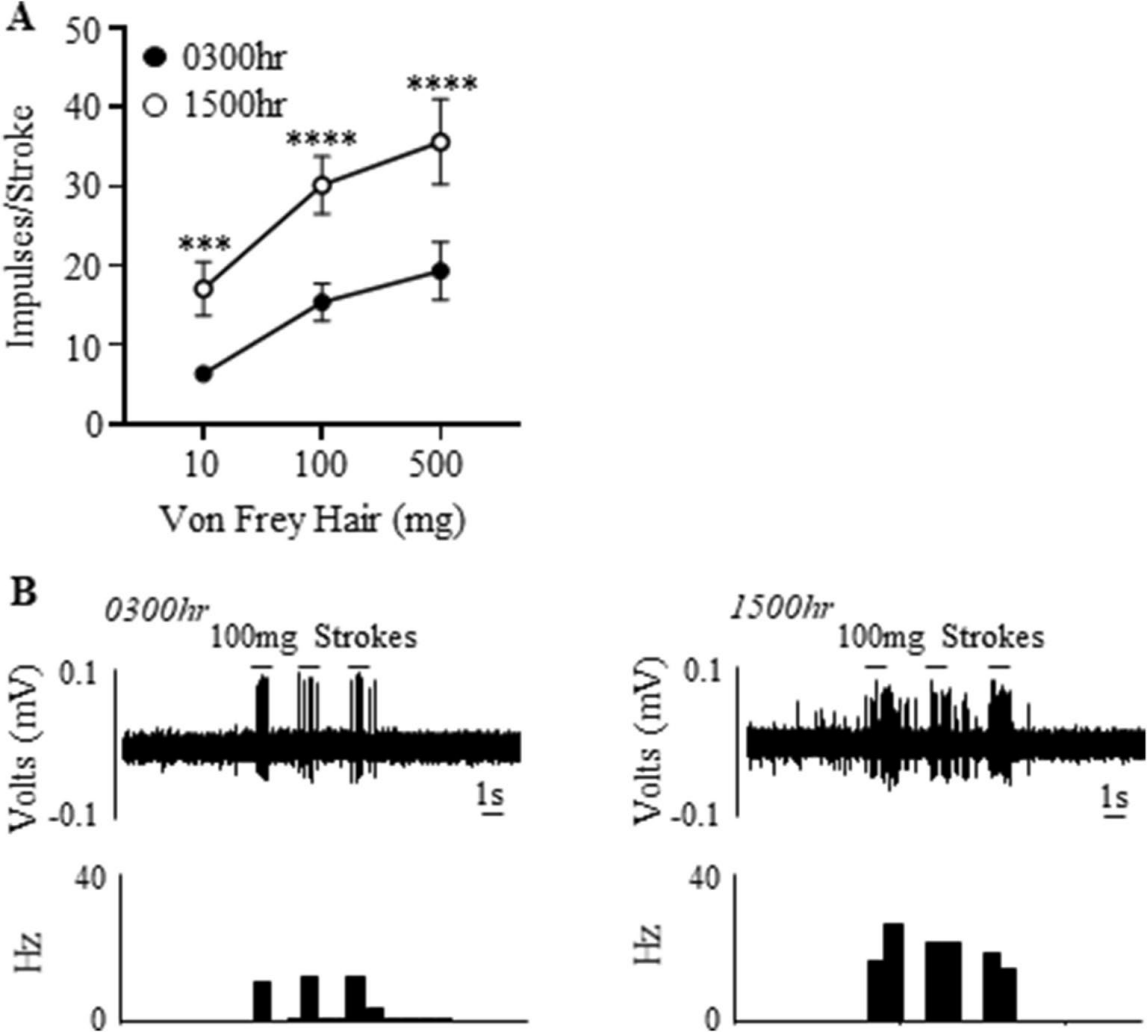
Bladder mucosal afferents are more mechano-sensitive during the day compared to the night. (**A**) Mucosal afferent response to mucosal stroking (10, 100, and 500 mg) at 0300 h and 1500 h. N = 6, n = 12 at each time point. ***P < 0.001, ****P < 0.0001. (**B**) An example of a raw trace of mucosal afferent responses to 100 mg stroking at 0300 h and 1500 h.

Time-of-day dependent effects on bladder low-threshold mucosal afferent mechano-sensitivity is illustrated in Fig. 4. Mucosal afferent sensitivity to stroking was significantly higher at 1500 h compared to 0300 h (Fig. 4A,B; time of day effect, (F(1,24) = 119.3, N = 6, n = 12, P < 0.0001)), stroking weight effect (F(2,24) = 53.6, P < 0.0001), and no interaction). This was more significant with increased stroke weights (Fig. 4A,B; 10 mg: 0300 h 6.3 ± 0.2 impulses, 1500 h 17.1 ± 1.5 impulses P < 0.001; 100 mg: 0300 h 15.4 ± 1.0 impulses, 1500 h 30.1 ± 1.6 impulses P < 0.0001; 500 mg: 0300 h 19.35 ± 1.6 impulses, 1500 h 35.6 ± 2.4 impulses P < 0.0001).

### Bladder mucosal afferents are more sensitive to N-oleoyl dopamine (endogenous TRPV1 agonist) during the day compared to the night

**Figure 5:**
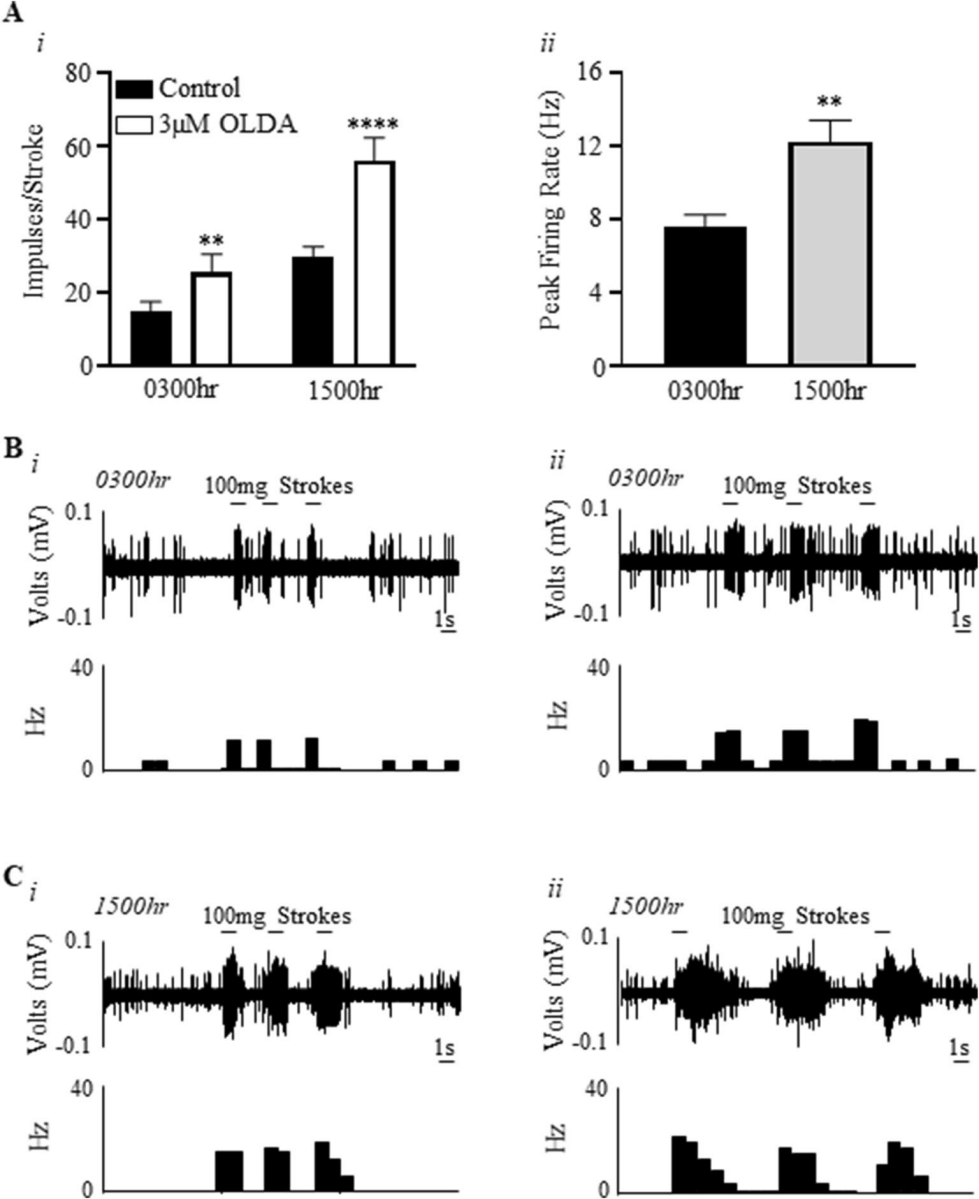
The response of mucosal afferents to N-oleoyl Dopamine is greater during the day compared to the night. (**Ai**) Mucosal afferent sensitivity to mucosal stroking (100 mg) before and after 3 μM N-Oleoyl Dopamine (OLDA) at 0300 h and 1500 h. (**Aii**) Direct activation of mucosal afferents by 3 μM OLDA at 0300 h and 1500 h. N = 6, n = 6. **P < 0.01, ****P < 0.0001. (**B**) An example of a raw trace of mucosal afferent responses to stroking in the (*i*) absence and (*ii*) presence of 3 μM OLDA at 0300 h. (**C**) An example of a raw trace of mucosal afferent responses to stroking in the (*i*) absence and (*ii*) presence of 3 μM OLDA at 1500 h.

The direct effect of endogenous TRPV1 agonist N-Oleoyl Dopamine (OLDA) on mucosal afferent activity and their mechano-sensitivity to stroking at each time point is illustrated in Fig. 5. OLDA significantly potentiated the response of mucosal afferents to stroking at both time points (Fig. 5Ai,B,C; drug effect (F(1,16) = 87.4, N = 6, n = 6, P < 0.0001), time-of-day effect (F1,16) = 133.4, P < 0.0001), and an interaction (F(1,16) = 15.79, P < 0.001). This response was significantly higher at 1500 h compared to 0300 h (0300 h: 73.1 ± 4.3%; 1500 h: 92.3 ± 7.1%, P < 0.05). The peak activation of mucosal afferents in response to OLDA was also significantly higher at 1500 h compared to 0300 h (Fig. 5Aii; 0300 h: 7.5 ± 0.6 Hz; 1500 h: 12.22 ± 1.2 Hz; N = 6, n = 6, P < 0.01).

## Discussion

The current study demonstrated for the first time a time-of-day dependent variation in bladder afferent mechano-sensitivity which may ultimately be involved in the control of voiding patterns. Further, this study also demonstrated time-of-day dependent variation in the response of bladder afferents to the endogenous TRPV1 agonist OLDA.

There is a complex hierarchical system in the neural control of micturition in humans and animals. Information about bladder filling is transmitted by bladder afferents to interneurons of the spinal cord which relay it to the periaqueductal grey, hypothalamus and cerebral cortex^9,10,26^. Inputs from the forebrain (prefrontal cortex) trigger conscious voiding by modulating the on–off switching brainstem neural circuitry which maintains a reciprocal function of the bladder and urethral outlet. It is believed that activity of bladder afferents (low or high level) is crucial for switching the reflex pathway from storage to voiding^10,27^. Voiding patterns differ dramatically between active and inactive phases in mice and humans^4–6^. The current data suggests that this too may be similar for guinea pigs, since they voided significantly more during their active phase (1500 h) compared to their inactive phase (0300 h), in agreement with a previous study in mice^5,6^. Interestingly, in guinea pigs, faecal pellet output also exhibits a diurnal variation pattern with significantly lower faecal pellet discharge during the night^28^. However, whether the day-night voiding pattern from the current study follows at other time points is not clear and requires further investigation across the full 24 h.

Urine storage and voiding is controlled by bladder afferents^8,10,29,30^. Both unmyelinated C fibres and thinly myelinated Aδ fibres innervate the bladder via sacral pelvic nerves and to a lesser extent lumbar splanchnic nerve^31,32^. For vagal afferent neurons there is clear evidence of a circadian rhythm modulation of gastric vagal afferent mechano-sensitivity which is inversely proportional to meal size^24,25^, suggesting a role in the circadian control of food intake. The current data for the first time suggests potential circadian rhythm modulation of the mechano-sensitivity of the spinal afferents innervating the urinary bladder in guinea pigs. The bladder afferents of at least three studied classes exhibit time-of-day dependent variation in their responses to mechanical stimuli. It is important to note that this was not due to changes in muscle tone since bladder wall compliance was not different between the day- and the night-time recordings. Since low and high-threshold muscular-mucosal afferents are likely involved in the signalling of bladder volume^11,33,34^, the reduced bladder afferent mechano-sensitivity during the night could allow a greater urine storage capability resulting in a smaller number of voids. Although this is consistent with the total voiding number and volume exhibited by the guinea pigs, the mean volume of each void did not differ between each of the measured timepoints. Given that bladder capacity is generally greater during rest periods^35,36^, it would be expected that volume per void during at 0300 h would be higher than 1500 h. However, a previous study in mice noted that the most significant changes in void volume occur immediately before and shortly after the light cycle changes phases^6^, further warranting the need to study voiding behaviours across a full 24 h period. It should be noted that urine production and secretion by the kidneys also follows a circadian rhythm^37^, and therefore the changes in voiding in the current study may also be related to this in addition to modulation of bladder afferent function. This requires further investigation.

High-responding mucosal afferents in the bladder are thought to play a role in the sensation of pain^11^, expressing ion channels, such as TRPV1, known to be involved in nociception. Circadian rhythm modulation of arthritic and neuropathic pain has been observed in rheumatoid arthritis and headaches^38^. Interestingly, TRPV1 itself is known to show a circadian rhythm in the bladder with expression peaking during active phases^22^. This is in agreement with the current data indicating a stronger direct excitatory response of mucosal mechanoreceptors to the endogenous TRPV1 agonist OLDA, and higher potentiation by OLDA of their mechano-sensitivity during the day compared to the night. This may partake in the control of potential bladder afferent rhythm and/or influence sensory processes such as discomfort and pain according to time-of-day. However, whether TRPV1 is controlled by circadian rhythms, or controls them is not clear since knockouts of TRPV1 result in loss of rhythms of some circadian oscillator genes such as *Bmal1* and *Per1* and *Per2*^39^. The role of circadian rhythms in bladder-related pain behaviours requires further separate investigation.

The mechanism for the time-of-day dependent changes in bladder afferent mechano-sensitivity is currently not clear. Circadian rhythms in the body are orchestrated by a central master clock located in the neuronal network of the SCN. At the cellular level of individual pacemaker SCN neurons, a complex transcription-translation feedback loop includes a set of oscillating ‘clock’ genes (e.g., *Clock*, *Nr1d1*, *Bmal1*, *Per1 *and *2*, and* Cry1* and* 2*). These are capable of regulating neuronal day-night differences in the firing rate of SCN neurons via the modification of expression and/or activity of several types Na^+^, Ca^2+^ and K^+^ channels^1,40^. Circadian clock genes are present in most cells and organs including the dorsal root ganglia (DRG)^41^, nodose ganglia^24,25^, spinal cord^42,43^, and bladder^5,6,20^. Therefore, it could be speculated that the rhythmic oscillation in the expression of clock genes in DRG may control the time-of-day dependent variation in bladder afferent mechano-sensitivity via modification of expression and/or activity of some ion channels. It has been previously shown that gastric vagal mechanoreceptors display a circadian rhythm^24^. However, high-fat diet-induced obesity abrogated the circadian variation in gastric vagal mechano-sensitivity without detectable changes in the circadian expression of clock genes in the whole nodose ganglia^25^. This may suggest other potential mechanisms for circadian control of visceral afferents. In the bladder, the expression of mechano-gated Piezo1 and TRPV4 channels in the mouse urothelial cells and TRPV1 in the rat bladder demonstrates clear circadian rhythms^19–22^. This suggests that changes in the expression of these ion channels may directly or indirectly, via release of ATP, influence the time-of-day dependent variation in bladder afferent mechano-sensitivity observed in the current study. This requires further investigation.

In summary, the current study demonstrates that three classes of bladder afferents, low- and high-threshold stretch-sensitive muscular-mucosal and low-threshold stretch-insensitive mucosal afferents, exhibit time-of-day dependent variation in mechano-sensitivity which may be related to day-night dependent changes in bladder function. Further studies across a 24 h period are warranted to reveal potential circadian rhythm modulation of bladder afferent mechano- and chemosensitivity.

## Methods

### Ethics

This study was approved by the Animal Welfare Committee of Flinders University (AEM1574-5) and conducted in accordance with the Australian Code for the Care and Use of Animals for Scientific Purposes (8th edition, 2013). This study also adhered to the ARRIVE guidelines^44^.

### Animals

Adult female guinea pigs (N = 18; weight 350–400 g; aged 5–6 weeks) were housed in a 12 h:12 h light: dark cycle with lights on at 0600 h and lights off at 1800 h, and with ad libitum access to a standard diet and water. These guinea pigs were used for experiments at two time points, 0300 h (night) and 1500 h (day) based on a previous study on feeding and faecal output^28^.

### Conscious voiding

To measure voiding number and volume, guinea pigs (N = 6) were housed in voiding metabolic cages for 3 h, 1.5 h each side of the selected time points. The animals were matched at each time point. Urine was collected in a 50 mL pot attached to a force transducer (Grass Force–displacement transducer FT03, Grass Instruments, Quincy, Mass, USA). It was assumed that 1 mL of urine was 1 g since the gravity of guinea pig urine is 1.015^45^.

### Ex vivo bladder afferent preparation

**Figure 6:**
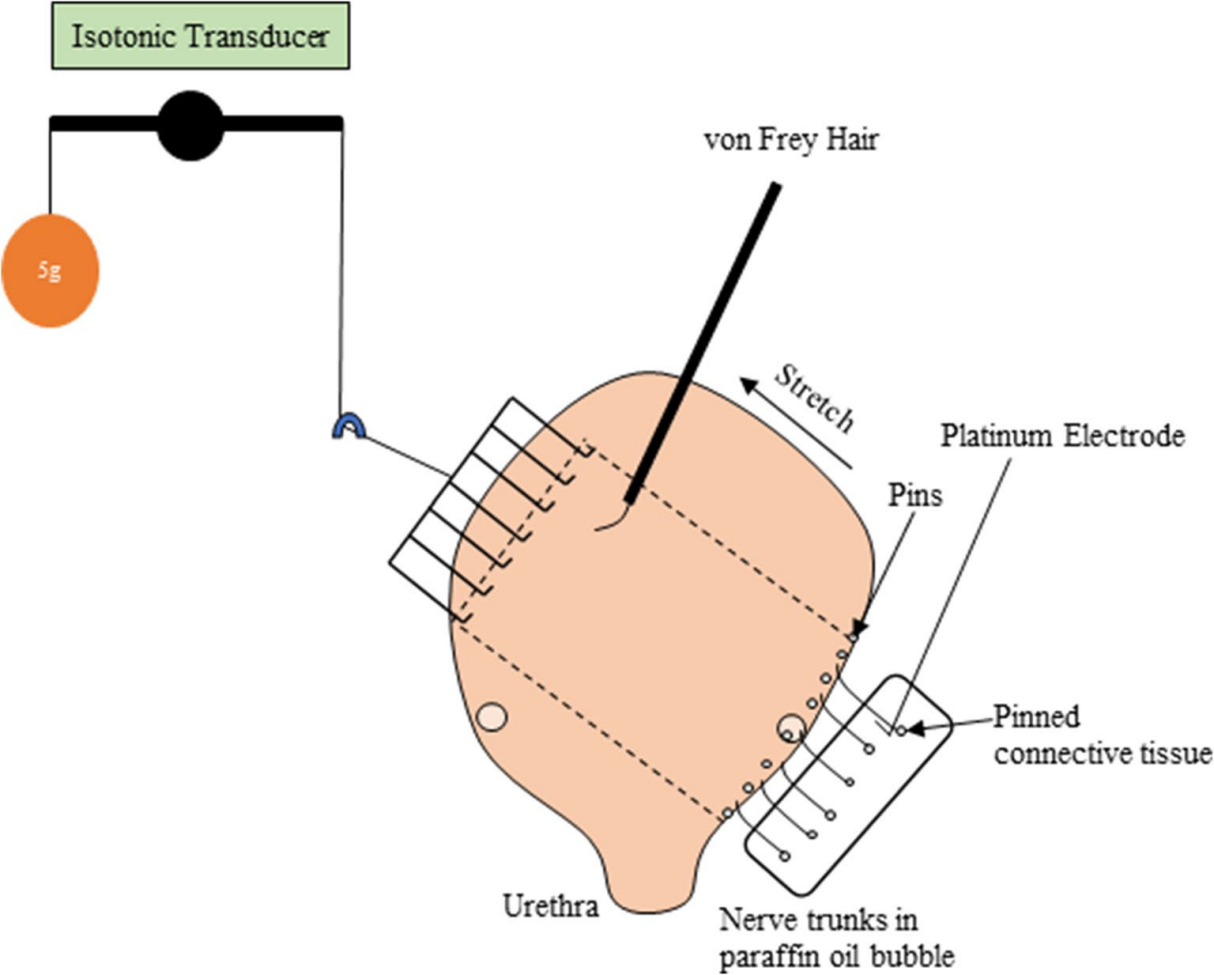
Schematic of the ex vivo extracellular recording set up. A region of bladder (approximately 12 mm wide by 15 mm long, shown schematically by the dashed lines) cut off the full thickness bladder flat-sheet preparation together with attached nerves. The nerve trunks are carefully dissected of the connective tissue and placed in a paraffin oil bubble. The opposite side of the bladder preparation is attached to a hook and cantilever system for stretch by placing the imposed loads (1–40 g) which is also connected to an isotonic transducer for measuring bladder lengthening. A von Frey hair is stroked across receptive fields in the mucosa. The nerve trunks are individually placed onto a platinum electrode for electrical recordings.

The animals were humanely culled via isoflurane overdose and cervical severing at 0300 h (N = 6) and 1500 h (N = 6). The ex vivo bladder afferent preparation for ‘close-to-target’ extracellular recordings of bladder afferent activity has been described in detail previously^12,34^. Briefly, the bladder and associated connective tissue containing nerve bundles was dissected out and opened along the midline of the anterior wall from the urethra up to the apex into a flat sheet in a modified Krebs solution consisting of (in mM): NaCl 118; KCl 4.74; NaH_2_PO_4_ 1.0 NaHCO_3_ 25; MgCl_2_ 1.2; CaCl_2_ 2.5; glucose 11 and nicardipine (3 µM) bubbled with 95% oxygen in 5% carbon dioxide. Several nerve trunks (4–7) entering the trigone area of the bladder between the left (or right) ureter and urethra were isolated from surrounding connective tissue. Then, a region of full thickness bladder (approximately 12 mm wide by 15 mm long, encompassing the bladder trigone and body) with attached nerves was created and pinned mucosal side up along one edge in a 22 mL organ bath continuously perfused with warmed 34 °C Krebs (3 mL/min). The nerves trunks were pinned loosely with 50 µm tungsten pins and individually placed on a platinum electrode in a paraffin oil bubble for electrical isolation and recording of electrical activity. The opposite edge of the bladder was attached via a hook and cantilever system, to an isotonic transducer (Harvard Bioscience 52-9511, S Natick, MA, USA) for stretching imposed by loads (1–40 g) and simultaneous measuring of the preparation lengthening (Fig. 6). A 30-min resting period was allowed before commencing experiments.

Electrical signals were amplified (DAM 80, WPI, USA), filtered via band-pass filter (BPF-932, CWE, USA, band pass 10 Hz–10 kHz) and recorded by a computer at 20 kHz with a Micro 1401-4 data acquisition system (CED, UK). Single units were discriminated offline by using Spike 2 software (version 10, CED, UK).

### Classes of bladder afferents

The current study focused on three classes of bladder afferents, mucosal high-responding, muscular-mucosal low threshold, and muscular-mucosal high threshold afferents. High responding mucosal afferents respond to light mucosal stroking but not to bladder stretch. This class is usually capsaicin sensitive^33,34,46^. Low-threshold muscular-mucosal afferents respond to both light stroking (10-100 mg) and stretch (1–5 g). High-threshold afferents respond significantly less to stretch. Usually, these sub-classes do not respond (or only fire a few action potentials) to stretch below 10–20 g in the guinea pig bladder^46,47^.

Afferent sensitivity to mucosal stroking was determined using calibrated von Frey hairs (10 mg) stroked across the receptive field at a rate of 5 mm s^−1^, 5 times. The middle three strokes were used for analysis. This was repeating with increasing intensities (100 mg and 500 mg). Afferent sensitivity to bladder stretch was determined by adding a 1 g weight to the cantilever for ten seconds. This was repeated with increasing weights (3, 5, 10, 20, 30, and 40 g) with a 1-min rest period between each weight.

### Effect of N-oleoyl dopamine on mucosal bladder afferents

Following the establishment of baseline bladder mucosal afferent sensitivity to stroking (100 mg), OLDA (3 µM; dissolved in ethanol, final concentration of ethanol in the bath was 0.1%) was added to the Krebs organ bath over the receptive field. The preparation was equilibrated for 5 min after which bladder mucosal afferent sensitivity was redetermined. In addition, OLDA-induced direct activation of mucosal afferents was analysed within 60 s of its application and peak firing rate was calculated by subtracting 10 s spontaneous firing (if any) from maximal firing during 10 s.

### Data analysis

All data is presented as the mean ± the SEM, with n referring to the number of afferents and N to the number of animals and analysed with GraphPad Prism 8 software. Voiding data was analysed with a paired t-test and bladder afferent data was analysed with a two-way ANOVA with Sidak’s post hoc test.
